# Diagnosis, Management and Outcome of Truncus Arteriosus Communis Diagnosed during Fetal Life—Cohort Study and Systematic Literature Review

**DOI:** 10.3390/jcm13206143

**Published:** 2024-10-15

**Authors:** Agnes Wittek, Ruben Plöger, Adeline Walter, Brigitte Strizek, Annegret Geipel, Ulrich Gembruch, Ricarda Neubauer, Florian Recker

**Affiliations:** Department of Obstetrics and Prenatal Medicine, University Hospital Bonn, Venusberg Campus 1, 53127 Bonn, Germany; agnes.wittek@ukbonn.de (A.W.); ruben.ploeger@t-online.de (R.P.); adeline.walter@ukbonn.de (A.W.); brigitte.strizek@ukbonn.de (B.S.); annegret.geipel@ukbonn.de (A.G.); ulrich.gembruch@ukbonn.de (U.G.); s4rineub@uni-bonn.de (R.N.)

**Keywords:** *Truncus* arteriosus communis, common arterial trunk, congenital heart defects, fetal echocardiography

## Abstract

**Background/Objectives**: Truncus arteriosus communis (TAC) is a rare congenital heart defect characterized by a single arterial trunk that supplies systemic, pulmonary, and coronary circulations. This defect, constituting approximately 1–4% of congenital heart diseases, poses significant challenges in prenatal diagnosis, management, and postnatal outcomes. **Methods**: A retrospective analysis was conducted at the local tertiary referral center on cases of TAC diagnosed prenatally between 2019 and 2024. Additionally, a systematic literature review was performed to evaluate the accuracy of prenatal diagnostics and the presence of associated anomalies in fetuses with TAC and compare already published data with the local results. The review included studies that especially described the use of fetal echocardiography, the course and outcome of affected pregnancies, and subsequent management strategies. **Results**: The analysis of local prenatal diagnoses revealed 14 cases. Of the 11 neonates who survived to birth, the TAC diagnosis was confirmed in 7 instances. With all seven neonates undergoing surgery, the intention-to-treat survival rate was 86%, and the overall survival rate was 55%. By reviewing published case series, a total of 823 TAC cases were included in the analysis, of which 576 were diagnosed prenatally and 247 postnatally. The presence of associated cardiac and extracardiac manifestations as well as genetic anomalies was common, with a 22q11 microdeletion identified in 27% of tested cases. **Conclusions**: Advances in prenatal imaging and early diagnosis have enhanced the management of TAC, allowing for the detailed planning of delivery and immediate postnatal care in specialized centers. The frequent association with genetic syndromes underscores the importance of genetic counseling in managing TAC. An early surgical intervention remains crucial for improving long-term outcomes, although the condition is still associated with significant risks. Long-term follow-up studies are essential to monitor potential complications and guide future management strategies. Overall, a coordinated multidisciplinary approach from prenatal diagnosis to postnatal care is essential for improving outcomes for individuals with TAC.

## 1. Introduction

Truncus arteriosus communis (TAC) also known as common arterial trunk, is a rare and complex congenital heart defect marked by one arterial trunk exiting the base of the heart in the absence of any atretic remnant of the aorta or pulmonary trunk; in the presence of the interventricular communication, a single arterial trunk emerging from the heart supplies systemic, pulmonary, and coronary circulations [1,2,3,4,5,6]. This defect is observed in approximately 1% to 4% of children with congenital heart disease, with an incidence of 9 to 11 cases per 100,000 live births. Although the etiology of TAC is believed to be multifactorial and it frequently occurs as an isolated anomaly, 22q11.2 microdeletion syndrome is identified in approximately 25% of patients with TAC [7]. The association is even stronger in cases with concurrent right-sided aortic arch, abnormal aortic arch branching patterns, and type B interrupted aortic arch. Three gene deletions on chromosome 22q11.2 (*TBX1*, *CRKL*, and *ERK2*) contribute to neural crest cell dysfunction and anomalies in the second heart field, leading to TAC and other defects associated with abnormal outflow tract cushion development. Other chromosomal abnormalities and gene mutations can also be associated with a TAC. Maternal diabetes has been recognized as an independent risk factor for TAC, with increased mortality observed in affected infants [6,8]. Furthermore, there is an elevated recurrence risk within families, including siblings, and reports of TAC occurring in dizygotic twins [8].

The prenatal diagnosis of TAC is crucial for optimal perinatal management. Advances in fetal echocardiography have significantly enhanced the ability to detect truncus arteriosus in utero and also allow the differentiation of its subtypes. The primary echocardiographic feature of TAC is the presence of a single arterial trunk overriding a large ventricular septal defect; cases of TAC without ventricular septal defect are absolutely rare [9]. Additional prenatal echocardiographic findings may include variable degrees of truncal valve insufficiency or stenosis as well as abnormalities of the pulmonary flow. Early and accurate diagnosis allows for comprehensive counseling of the parents regarding the prognosis and management options as well as the planning of delivery in a tertiary care center equipped with specialized neonatal and cardiothoracic surgical services.

Management strategies during affected pregnancies focus on monitoring fetal growth and cardiac function. Regular follow-up with fetal echocardiography is essential to assess the progression of the defect and to identify associated anomalies or complications, such as truncal valve dysfunction (stenosis and/or insufficiency) or hydrops fetalis [10]. In some cases, maternal medical therapy may be indicated to optimize fetal hemodynamics. Delivery planning involves multidisciplinary coordination to ensure immediate postnatal stabilization and transfer to a specialized cardiac center.

Postnatal management of infants with TAC typically involves prompt stabilization and balancing blood flow through pulmonary and systemic circuits, including the administration of prostaglandin E1 in cases showing the Van Praagh subtypes A3 and A4 [2] to maintain ductal patency and ensure adequate systemic and pulmonary blood flow, respectively. Pulmonary congestive heart failure may require the use of diuretics and in the case of respiratory distress, respiratory support too. Early surgical intervention is imperative, usually within the first few weeks of life, to prevent irreversible damage to the pulmonary vasculature and further complications. The primary surgical procedure performed as definitive correction involves the repair of the ventricular septal defect as well as the separation of the pulmonary arteries from the truncus arteriosus. Therefore, a conduit from the right ventricle to the pulmonary arteries is created by different techniques and materials without or rarely with a valve. Abnormalities of the aortic arch and the truncal valve may also be fixed [6,11].

The surgical outcomes for TAC have improved significantly with advances in cardiopulmonary bypass techniques, perioperative care, and postoperative management. However, the condition remains associated with considerable risks, including perioperative mortality, late mortality predominately during the first year of life, and residual cardiac lesions. Furthermore, approximately 75% of patients require re-interventions due to the likelihood of outgrowing their right ventricular pulmonary artery conduit or experiencing a worsening of truncal valve insufficiency [12].

The purpose of this study was to review the diagnosis, management, and outcome of local TAC cases as well as published case series to determine the accuracy of prenatal imaging in reaching a correct diagnosis and detecting the presence of associated anomalies.

## 2. Materials and Methods

### 2.1. Data Collection of Local Cases

A retrospective analysis was conducted on all cases of TAC prenatally diagnosed at a German tertiary referral center (University Hospital Bonn) between 2019 and 2024. The analysis focused on the outcome of the pregnancy, pediatric surgeries, and overall survival. The data collection of the cases was performed using the local hospital’s internal database. Following an initial keyword search for the diagnosis of TAC, cases with a prenatal diagnosis were identified and included. Cases diagnosed prior to 2019 were already covered in a multicenter analysis by Abel et al. and thus were not included in this analysis to avoid duplication of data [13]. Neonates with a postnatal diagnosis other than TAC were excluded from further analysis. Pregnant women whose fetuses were suspected of having TAC and whose data were included in this analysis provided informed consent for the utilization of their data for scientific evaluation.

### 2.2. Literature Review and Analysis

The studies were subjected to a rigorous and comprehensive evaluation to ensure a systematic assessment. Case series reporting at least 10 cases of prenatally diagnosed TAC with information on the course, management, and outcome of the pregnancy and neonatal period as well as the imaging assessment methodology employed for diagnosing and evaluating TAC were included. The literature search covered multiple databases to ensure a thorough review of the existing literature. The databases searched included PubMed, Medline (covering the period from 1950 to May 2024), and Embase (from 1980 to May 2024). Additionally, the Cochrane Library and the Web of Science with Conference Proceedings (from 1970 to 2024) were also included in the search. To further ensure that no relevant studies were overlooked, the reference lists of pertinent articles were reviewed. The search strategy was not limited by language, allowing for the inclusion of studies published in multiple languages, thus broadening the scope of the review. Moreover, auto-alerts in Medline were employed throughout the review process to identify any newly published studies that met the inclusion criteria.

Three authors, identified as A.W., U.G., and F.R., undertook an independent review of all abstracts. This process was crucial for maintaining objectivity and reducing bias. Each abstract was scrutinized meticulously, and the authors reached a consensus regarding the potential relevance of each study. When agreement was achieved, full-text copies of the relevant papers were obtained for a more detailed review. The reviewers then independently extracted relevant data from these full-text papers focusing on study characteristics, such as the design, sample size, and methodology as well as on pregnancy outcomes. To ensure consistency and accuracy, any discrepancies or inconsistencies in the data extraction process were thoroughly discussed among the reviewers. In cases where consensus could not be reached among the three reviewers, a fourth author was consulted to resolve the issues, ensuring that a final consensus was achieved.

To avoid the inclusion of duplicate data, particularly when multiple studies were published on the same cohort with identical endpoints, a stringent selection process was employed. The report containing the most comprehensive information on the population was included to prevent the overlap of data and to ensure that the analysis was based on the most complete dataset available.

### 2.3. Methodological Evaluation of Included Case Series

In order to evaluate the methodological conduct and reporting of the included case studies, an assessment system designed by Murad et al. was used, combining the criteria of Pierson, Bradford Hill, and the Newcastle–Ottawa [14]. The concept proposes guiding questions for the methodological evaluation of case reports and series which were applied to the subjects of this study (see Table 1) [15]. Firstly, the selection criterion examines whether the inclusion of potential patients is comprehensive and complete. The ascertainment category assesses the reliability of the collected data and outcomes based on the sources used, such as medical files and clinic databases or patient interviews. Furthermore, the causality item evaluates whether the observation period for the patients was sufficient to determine the extent of the disease and its outcomes. The quality of reporting is measured by how well the research can be replicated based on the presented methodology and whether the results provide relevant added value for clinical practice.

### 2.4. Analysis of Included Studies and Data Synthesis

The case series included in the study were analyzed according to the number of cases prenatally and postnatally diagnosed with TAC as well as the outcome of the affected pregnancy and neonate. The relevant data from the studies were extracted, collected, and analyzed using Microsoft Excel Version 16.78.3 (Microsoft Co., Washington, DC, USA). Summarized data of the reviewed literature was then compared to the number and outcome of local TAC cases. In the following, results were tabulated using Microsoft Word Version 16.78.3. In the event of a lack of available information, this is indicated in the tables.

### 2.5. Classification of Morphological TAC Subtypes

#### 2.5.1. Collett and Edwards (1949)

Over the last decades, several attempts have been made to classify the different morphological variants of a common arterial trunk into different subtypes. The first attempt to establish a classification system was made by Collet and Edwards in 1949 [1]. They defined four different subtypes based on the presence and location of the pulmonary arteries (PAs). Common arterial trunks with present PAs are categorized into types 1–3. Type 1 still shows a short aortopulmonary septum resulting in an interposition of a short main pulmonary artery, from which both PAs arise. In type 2 and 3, a pulmonary trunk is no longer present and both PAs directly arise from the common arterial trunk. The outlets of the PA are located either close to each other on the dorsal truncus (type 2) or on the opposite sides of the truncus communis (type 3). The case of absent PAs, in which pulmonary perfusion is maintained via aortopulmonary collaterals of the bronchial arteries, was originally defined by Collet and Edwards as type 4 [1]. Nowadays it is no longer considered a subtype of common arterial trunk, but rather a variant of pulmonary atresia with a ventricular septal defect. However, this initial classification does not take into account the aspect of aortic or pulmonary dominance.

#### 2.5.2. Original Van Praagh Classification (1964)

However, as the absence of one PA can have a significant influence on the prognosis and clinical presentation, a revised alphanumeric classification was introduced by Van Praagh in 1964. Furthermore, it also considers the cardio-surgical outcome of the subtypes, namely the poor outcome in the presence of an interrupted aortic arch [2]. While type 1 is the same in both classification systems, Van Praagh et al. combine types 2–3 of Collet and Edwards into one (Van Praagh type 2) [2]. In type 3, Van Praagh et al. describe a morphological variant in which one PA arises from the common arterial trunk and provides blood to one side of the lung, while the other PA origins extrapericardially by a patent arterial duct. In addition, the authors supplemented the classification of Collet and Edwards with Van Praagh type 4, also taking into account variants of the aortic arch such as a severe coarctation or interruption of the aortic arch. Furthermore, they labeled the four types with the letter A (type A1–A4) in the presence of interventricular communication and correspondingly with the letter B (type B1–B4) in the very rare cases of a TAC without ventricular septal defect.

#### 2.5.3. Modified Van Praagh Classification (2000)

Considering inconsistencies in determining the exact location of the PA on the common arterial trunk, the initial Van Praagh classification was first simplified by Calder et al. in 1976 and later by Jacobs and the members of the Congenital Heart Surgery Database Committee and European Association for Cardiothoracic Surgery in 2000 based on the concept of either aortic or pulmonary dominance of the intrapericardial part of the common arterial trunk [3,4]. Van Praagh A1 and A2, respectively types 1, 2, and 3 according to Collet and Edwards, were merged into one subtype characterized by confluent or near-confluent PAs (large aorta type) [4]. As the other defined types correspond to Van Praagh A3 (large aorta type with only one PA and supply of the other PA by a patent ductus arteriosus) and Van Praagh A4 (large PA type and aortic hypoplasia), this classification is also referred to as modified Van Praagh Classification.

#### 2.5.4. Russell et al. (2011)

A classification proposed by Russell et al. in 2011 narrows the subtypes to a differentiation into an aortic or pulmonary dominance [5]. If both or at least one PA originates from the common arterial trunk, aortic dominance is present. In the case of hypoplastic or interrupted aortic arch and coarctation, there are a discrete pulmonary trunk and a hypoplastic aorta within the pericardial cavity and the supply of the systemic downstream circulation is dependent on a patent arterial trunk; the corresponding type is referred to as pulmonary dominant. The autopsy of 28 fetal hearts with a common arterial trunk revealed that opposite originating PAs, as initially described by Collet and Edwards in type 3, only manifest in cases of pulmonary dominance. On the contrary, in the case of aortic dominance, outflow patterns of the PAs can be classified into further subtypes, as their location is often essential for surgical planning. This further simplification emphasizes important morphologies as the hypoplastic aortic arch of the pulmonary dominant type often plays a decisive role in the surgical outcome and mortality. As the great majority of TAC cases show a defect of the ventricular septum, this classification also renounces the alphanumeric distinction in the A and B subtypes proposed by Van Praagh et al. [2].

A consensus paper by the European Association for Cardio-Thoracic Surgery (EACTS) and the Association for European Pediatric and Congenital Cardiology (AEPC) on the management of patients with TAC recommends the approach by Russell et al. for future morphological categorizations [5]. Nevertheless, as all analyzed cases were still diagnosed using previous classification systems and in order to consider different PA patterns, the following analysis employs the modified Van Praagh classification [4].

#### 2.5.5. Statistical Analysis

Statistical analysis was performed with Microsoft Excel^®^ (Version 16.78.3, Redmond, WA, USA). Mean, median, standard deviation (SD), range, and 95% confidence intervals (CI) were calculated. Data are shown as numbers and percentages (n and %, respectively).

## 3. Results

### 3.1. Analysis of Local TAC Cases

**Figure 1:**
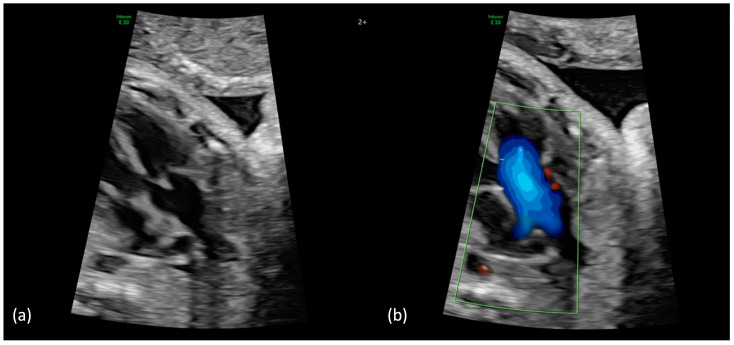
(**a**,**b**): Truncus arteriosus type 1 at 31 + 4 weeks. (**a**) Perimembranous outlet VSD as interventricular communication with the broad common arterial trunk in the overriding position. (**b**) In the color Doppler mode, the filling of the common arterial trunk from both ventricles and the posterior branch of one pulmonary artery can be seen (aortic dominance); aliasing or a high variance, which indicates a stenosis of the truncal valve, is not present.

**Figure 2:**
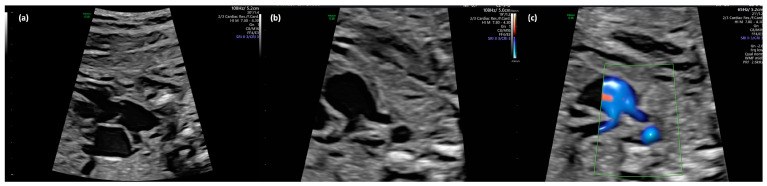
(**a**–**c**): Truncus arteriosus type 1 at 22 + 0 weeks in B-mode (**a**,**b**) und with color doppler (**c**).

**Figure 3:**
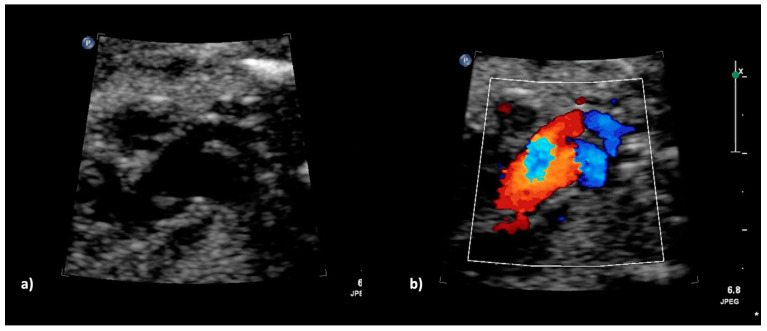
(**a**,**b**): Truncus arteriosus type 4 with interrupted aortic arch type b at 23 + 5 weeks. (**a**) The 2DE shows the ventricular outlet part with the overriding common trunk, the truncal valve as well as the wide truncus pulmonalis and the hypoplastic ascending aorta (pulmonary dominance); (**b**) The color Doppler mode shows the outflow from both ventricles into the common arterial trunk and further into the pulmonary trunk as well as into the hypoplastic aorta.

**Figure 4:**
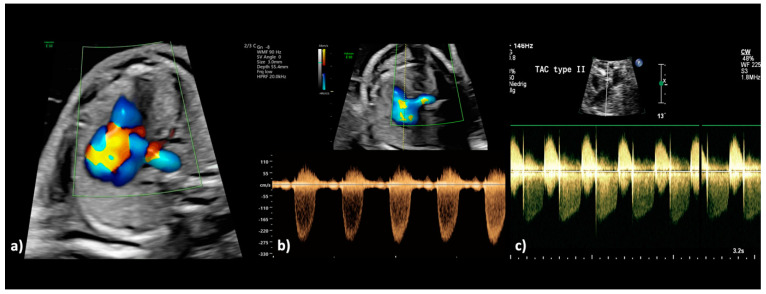
(**a**–**c**): Truncus arteriosus type 1 at 22 + 0 weeks. (**a**) In the color Doppler mode, the filling of the common arterial trunk from both ventricles and the confluent origin of both pulmonary arteries from the posterior side of the aorta is visualized (aortic dominance), as well as aliasing and high variance as indications of disturbed blood flow due to stenosis of the truncal valve. (**b**) The spectral Doppler examination confirms the presence of a stenotic truncal valve by a high maximum velocity (peak systolic velocity: 3.0 m/s; maximum pressure gradient (ΔPmax: 36 mm Hg) and wide variation of the blood flow. (**c**) In another fetus with truncus arteriosus type A2 at 34 + 1 weeks, there is both a relevant stenosis and a severe regurgitation of the truncal valve; during systole, the tubular antegrade flow with a peak velocity of 2.40 m/s can be recognized; during diastole, the insufficiency of the truncal valve with a peak velocity of 3.30 m/s can be recognized.

The investigation of the clinic’s internal database yielded 14 cases of prenatal diagnoses of TAC (see Table 2). The mean gestational age of the pregnancies at the time of referral was 22 + 3 weeks (range: 16 + 2 to 33 + 6 weeks). During the course of the pregnancy, the parents elected to end the pregnancy in four cases. The mean gestational age at the time of termination of the pregnancy was 20 + 4 weeks (range: 19 + 2 weeks to 25 + 3 weeks). No cases of spontaneous intrauterine fetal demise were observed. A total of 10 fetuses were born alive, 5 of whom were delivered by primary or secondary cesarean section and 5 by vaginal delivery, one of which was delivered by vacuum extraction. The mean gestational age at the time of delivery was 37 + 6 weeks (range: 34 + 2 to 39 + 5 weeks). Of 10 live births, the diagnosis was revised to other severe congenital heart defects in 3 cases (cases 5, 9, and 12). In one case a rare diagnosis with one great right sided MAPCA arising from DAO and perfusion of the LPA via DA from the aortic arch with RAA was diagnosed. Therefore, regarding postnatally confirmed diagnoses, the false-positive rate of fetal echocardiography in this cohort was 30%. No autopsies were performed on the terminated pregnancies. With regard to the classification of large vessel morphology in accordance with the modified Van Praagh scheme, the subtype described was modified in two out of seven of the confirmed TAC cases. Only a small number of cases underwent prenatal genetic analysis. Notably, there were no instances of microdeletion 22q11 in our cohort, however, one case did present with trisomy 13. The majority of cases were associated with a variety of cardiac and extracardiac malformations, which exhibited considerable variability. However, malformations frequently associated with TAC, such as a right aortic arch or abnormalities of the truncal valve or atrioventricular valves, were also observed recurrently in this cohort (Figure 1, Figure 2, Figure 3 and Figure 4). All seven live births underwent surgical intervention. One neonate died on the fifth postoperative day following the binding of the pulmonary arteries. The remaining children were alive at the time of the final follow-up. The mean follow-up period was 30.5 months (range: 8 days to 56 months).

### 3.2. Review and Comparison with Published Case Series

#### Selection and Evaluation of Methodological Quality of Included Case Series in the Literature

**Figure 5:**
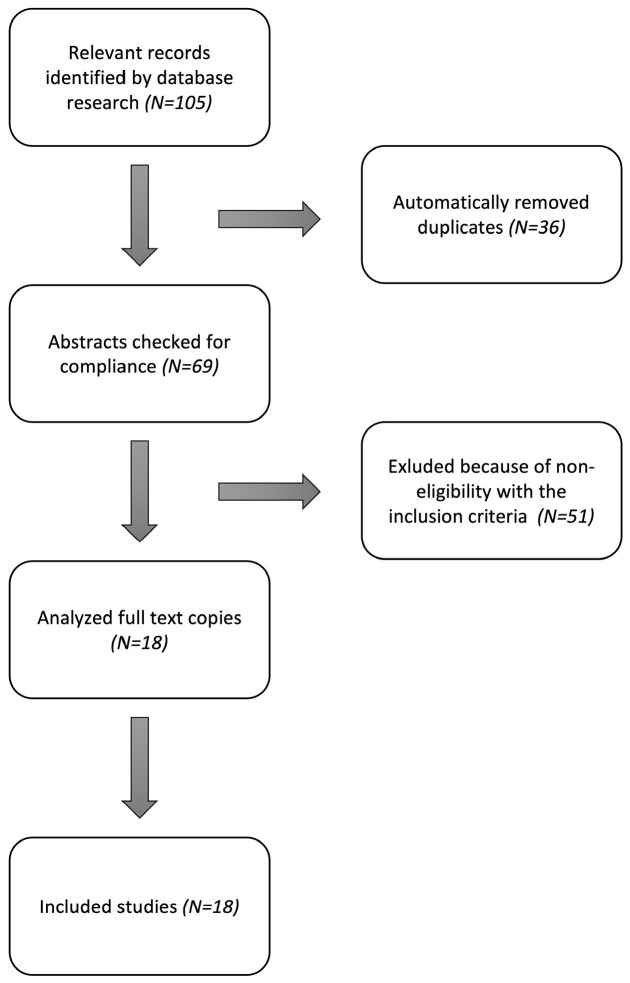
Literature selection process according to PRISMA guidelines.

The literature search initially identified 105 relevant publications. As a result of the subsequent careful review, 18 case series were included in the analysis (see Figure 5).

Overall, most studies fully met the evaluation criteria according to Murad et al. (see Table 3) [14]. Each study specified a data collection period during which they retrospectively included all patients with suspected TAC pregnancies. In all studies, the data collection on the presence and outcome of a TAC was based on internal databases of the study centers, e.g., encompassing documented echo examinations, birth reports, and surgical reports. All included studies are retrospective studies, analyzing the data of their local center (and referring clinics). Each study followed the cases up to the induced or natural end of pregnancy or birth and for a postnatal period.

### 3.3. Study Characteristics of Reviewed Literature

In general, the data cover an average observation period of 11 years (range: 5–24 years) between 1990 and 2024 (see Table 3). Regarding the timing of the initial diagnoses, 11 studies only included cases that were diagnosed by fetal echocardiography during pregnancy while the other 7 also looked at the outcome of postnatally diagnosed children. Overall, the reviewed case series add up to a total of 823 cases of TAC, of which 576 (70%) were diagnosed by fetal echocardiography and 247 (30%) postnatally. Most of the studies pertain to European or North American populations, with only one study examining TAC cases in La Réunion and two studies from Chinese and South Korean centers, respectively.

### 3.4. Diagnostic Accuracy and Pregnancy Outcome of Fetal Echocardiography

The average gestational age at referral to the respective obstetrical department and subsequent diagnose of TAC in the reviewed studies was 23 ± 2 weeks, ranging from 12 to 40 weeks (see Table 3). Most studies analyzed the accuracy of fetal echocardiography in diagnosing TAC. Determined by the proportion of postnatally confirmed cases out of the total suspected prenatal diagnoses, fetal echocardiography had a median accuracy of 82% ranging from 6% to 100% in different studies and study periods. Several studies have examined the benefits and value of fetal echocardiography in TAC over time, all demonstrating an increase in both diagnostic accuracy and the number of diagnoses made using this imaging technique [9,18,19,26]. For instance, Morgan et al. reported a diagnostic accuracy of 6% for the period from 1990 to 1999, which rose to 45% between 2000 and 2014 [26]. Evans et al. even recorded an increase in diagnostic accuracy from 33% during the data collection period of 2006–2009 to 93% between 2010 and 2021 [19]. Out of 576 suspected fetal cases identified prenatally, the parents of 206 (35.8%) decided to terminate the pregnancy. In total, 26 cases (4.5%) were documented in which spontaneous intrauterine fetal death occurred. Ultimately, a total of 342 (59.3%) live births were recorded.

### 3.5. Classification Systems

**Figure 6:**
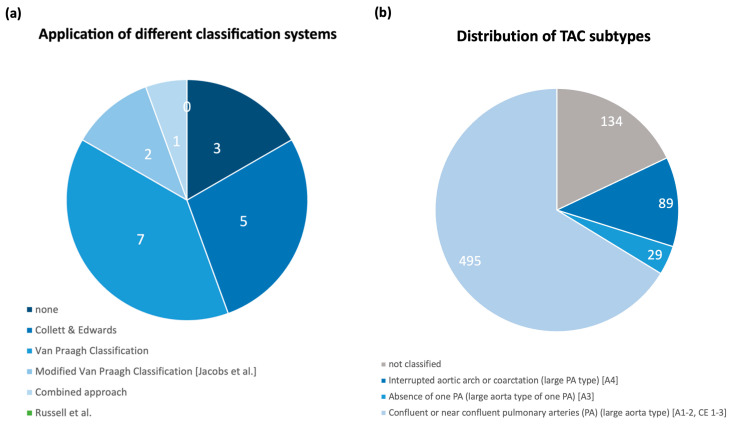
**Classification of TAC subtypes.** (**a**) Use of different classification systems by the included case series. (**b**) Number of different TAC subtypes in reviewed studies (including the cases of this study) using the modified Van Praagh classification in 2000 [9]. **Large aorta type (A1–2)**: TAC with confluent or near confluent pulmonary arteries (PA); **large aortic type of one PA (A3)**: TAC with absence of one unilateral proximal PA and the presence of ductus arteriosus or collateral arteries, which supply the lungs in the absence of a pulmonary artery branch from the truncus; **large pulmonary type (A4):** TAC with aortic hypoplasia resulting from interrupted aortic arch or severe aortic coarctation; A1–A4 refers to original Van Praagh classification [2], CE = Collet and Edwards, Types 1–3 [1].

Various classification systems were used to categorize the severity of TAC, with most studies applying either the conventional Van Praagh classification, the modified Van Praagh system according to Jacobs et al., or the classification system proposed by Collet and Edwards [1,2,4]. Abel et al. used a combined approach of both classifications [13]. None of the analyzed case series applied the latest classification published by Russel et al. into two subtypes according to aortic or pulmonary dominance. Three studies did not specify the anatomical subtypes of their reported cases (see Figure 6a) [9,16,27]. Figure 6b shows the distribution of the classified cases of all reviewed studies to the subtypes defined by the modified Van Praagh classification.

**Figure 7:**
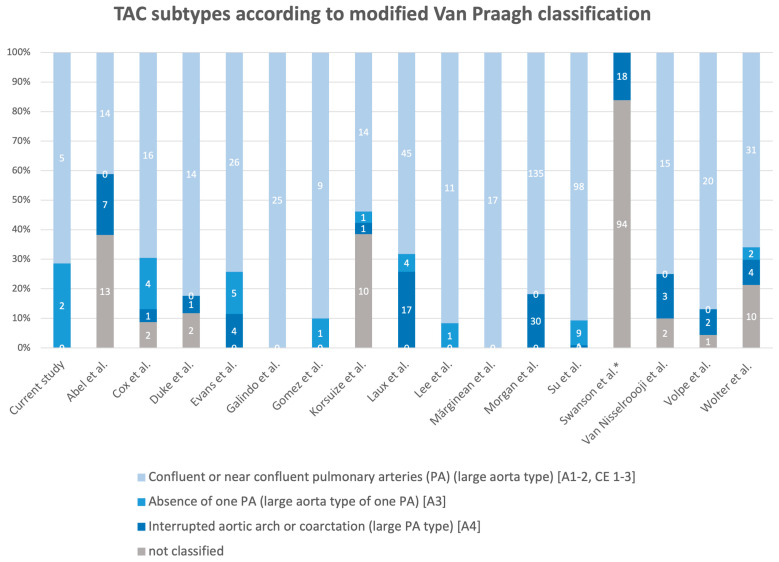
Assignment of TAC cases in the included studies [9,13,17,18,19,20,21,22,23,24,25,26,28,29,30,31] to the three subtypes of the modified Van Praagh classification [4]: type A1–2: TAC with confluent or near confluent pulmonary arteries (PA) (large aorta type); type A3: TAC with absence of one PA (large aorta type of one PA), the other PA supplied by patent arterial duct); type A4: TAC with interrupted aortic arch or coarctation (large PA type). A1–A4 refers to the original Van Praagh classification [2], CE = Collet and Edwards, types 1–3 [1]. * does not represent all cases, other subtypes were not specified.

For 613 cases of TAC, the morphological arrangement of the great vessels was described using a subtype of one of the common classification systems. By transferring the different approaches into one classification, a total of 495 (80.7%) cases corresponded to the large aorta type, with confluent or near confluent PA arising from the common arterial trunk. This type unifies the Van Praagh types A1 and A2 as well as types 1–3 according to the classification proposed by Collet and Edwards. A total of 29 cases (4.7%) could be assigned to the large aortic type with the absence of one unilateral proximal PA and the presence of ductus arteriosus or collateral arteries, which supply the lungs in the absence of a pulmonary artery branch from the truncus, also documented as Van Praagh A3 in the literature. A total of 89 cases (14.5%) were found to have hypoplastic aorta in the presence of interruption or severe preductal coarctation and were, therefore, classified as a large PA type (corresponds to Van Praagh A4). In 134 cases (21.8%) the anatomical subtype was not specified. In addition to assessing the overall diagnostic accuracy of fetal echocardiography at their local center, six studies also examined the success rate of sonographic classification into the correct anatomical subtypes. Ranging from 80.3–100%, the average diagnostic accuracy was 90.25% (SD ± 13.5) (see Table 3). Figure 7 shows the distribution of TAC subtypes among each case series.

### 3.6. Associated Truncal Valve Regurgitation or Stenosis

As comorbidities potentially influence surgical outcomes and the prognosis of the affected children, further cardiac and extracardiac anomalies were also reported by most studies. Adding cases from the literature and our local cases, at least 162 cases of the analyzed patients showed an isolated TAC (TAC cases with no genetic diagnosis or any extracardiac manifestation), making up for an average proportion of 41% in the reported studies. Commonly recorded further cardiac conditions were defects of the truncal valve, documented in 186 cases. With regard to the pre- and postnatal echocardiographic comparison of truncal valve function, an analysis of isolated cases of TAC with truncal valve dysplasia showed that postnatal evaluation confirmed the prenatal findings in most cases—in two cases, a stenosis or regurgitation was found postnatally that was not diagnosed on fetal echocardiography (see Table 4). Of the 11 pregnancies that were delivered, 2 neonates died before surgery. The remaining nine neonates were all operated on, and seven were alive at the latest follow-up.

### 3.7. Associated Extracardiac Anomalies

Ranging from 17% to 80%, the proportion of children with additional extracardiac anomalies affecting other organ systems varied greatly. Observed extracardiac involvements included thymus aplasia, hygroma colli, cerebellar hypoplasia, microcephaly, singular uterine artery, and colonic atresia as well as limb anomalies. In addition, prenatal cases have also presented with intrauterine growth restriction and polyhydramnios. Comparing the proportion of children with extracardiac anomalies within the antenatal and postnatal cohort, Swanson et al. noted a higher incidence of extracardiac anomalies among prenatally diagnosed cases [9]. The authors attributed this to the fact that extracardiac anomalies lead to a more in-depth sonographic examination of the fetus and thus also to a higher probability that congenital heart defects are detected earlier than in extracardiac inconspicuous fetuses [9]. In the study by Morgan et al., the live-born patients of the cohort diagnosed during the fetal period showed no differences in the severity of cardiac pathology and postnatal management compared to the postnatally diagnosed group. However, they were born significantly earlier (37 weeks vs. 39 weeks gestational age, *p* < 0.001) and had a lower birth weight (2.56 kg vs. 2.87 kg, *p* = 0.01) [26].

### 3.8. Genetic Analysis

The occurrence of TAC is associated with an increased prevalence of genetic anomalies. In total, 525 cases underwent further genetic analysis. The methods used to obtain the sample material and the level of genetic analysis (karyotyping or further array analyses) certainly differed, also taking into account the very different periods of data collection.

Chromosomal abnormalities of any kind were found in 181 (34%) cases. Most commonly, a 22q11 microdeletion was present in 143 cases (27%). Additionally, 77 cases (15%) exhibited other numerical or structural chromosomal abnormalities including trisomies of chromosomes 9, 13, 18, and 21 as well as mosaic-types of trisomy 16 and 9, with trisomy 13 being the most frequent. Structural chromosomal abnormalities were also identified, such as different patterns of deletions and duplications (see Table 5). Three studies also examined the family histories of TAC fetuses, identifying a proportion of 6/17 (35%), 3/23 (13%), and 4/23 (17%) cases, respectively, with a positive family history of congenital heart diseases [17,18,30].

### 3.9. Termination of Pregnancy

The rate of termination of pregnancy following a diagnosis of TAC is high, although it varies greatly over the years and between countries between 10% and 40% (Table 3). Often the association with genetic and other extracardiac anomalies leads to the parental decision to terminate the pregnancy. The extent to which the presence of an interrupted aortic arch or an incompetent truncal valve favors a termination cannot be determined from the prenatal series.

### 3.10. Outcome and Survival

The continued pregnancies of prenatal suspected cases resulted in a total of 342 live births. Additionally, 247 children were postnatally diagnosed. Out of a total number of 589 live births diagnosed with TAC, at least 53 children died during the early neonatal phase before they could be surgically treated (pre-OP-neonatal death, pre-OP-NND, see Table 6). Primary repair of the heart defect was attempted for the majority of the patients affected. Only 16 patients were treated palliatively. A total of 60 patients died shortly after the operation, representing 12.5% of the 478 cases that underwent surgery. Taking into account the widely differing periods of follow-up, 219 patients were alive at the time of the last data collection, which corresponded to an average of almost 55% in the individual studies.

Laux et al. and Swanson et al. analyzed the differences in outcomes between prenatally and postnatally diagnosed TAC cases and found that prenatally diagnosed patients underwent surgery earlier and at a lower body weight [9,23]. Considering the shorter preoperative phase for these patients, there was no significant difference in preoperative mortality between the prenatally and postnatally diagnosed TAC groups. Furthermore, perioperative management showed no differences regarding the need for non-invasive ventilation or intubation, the occurrence of acidosis, acute heart failure or shock, or the need for extracorporeal ventilation and intensive care. However, the duration of intubation and the total length of hospitalization were significantly shorter for prenatally diagnosed cases [23]. Additionally, a Kaplan–Meier analysis with a median follow-up period of 2.24 years revealed no differences in overall mortality between the groups [23]. On the contrary, Swanson et al. came to different conclusions. In their study, the proportion of early postoperative deaths in the prenatal cohort was three times as high as in the postnatally diagnosed cases [9]. Regarding the complexity of TAC, the study by Morgan et al. demonstrated that cases of simple TAC were operated on significantly later [26]. The comparison of the outcome of TAC patients over time between 1990 and 2014 showed a continuous improvement in survival rates [26]. Before 2000, the survival rates after one month, one year, and 10 years were 66%, 54%, and 50% respectively. These rates increased to 93%, 85%, and 81% after 2000. In addition, operative mortality decreased from 36% to 7% (20 of 56 versus 5 of 74; *p* < 0.0001) [26].

**Figure 8:**
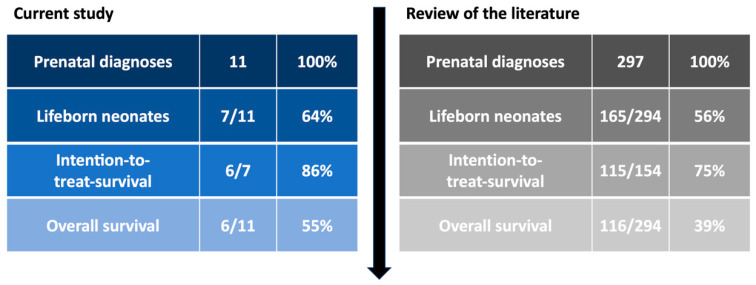
**Comparison of overall survival of prenatally diagnosed TAC cases of the current study and reported case series:** In the current study, 64% of prenatally diagnosed cases resulted in live births, with an 86% intention-to-treat survival and 55% overall survival. The literature review shows 56% live births, 75% intention-to-treat survival, and 39% overall survival, indicating better survival rates in the current study.

In addition to the data from the current study, nine case series were identified that specifically investigated the outcome of prenatally diagnosed cases (see Table 7). Including neonatal death pre- and postoperatively, as well as cases that were treated with a palliative treatment goal, it was possible to draw conclusions about intention-to-treat and overall survival from the data from these studies. The observed rates were somewhat higher in our local data set than in the overall evaluation of the literature, although the relatively small number of cases must be acknowledged as a potential limitation (see Figure 8).

## 4. Discussion

Truncus arteriosus communis (TAC) is a rare and complex congenital heart defect characterized by a single arterial trunk arising from the heart that supplies systemic, pulmonary, and coronary circulations. Despite accounting for only 1–4% of all congenital heart defects, TAC poses significant challenges in both prenatal and postnatal care due to its complex anatomy and associated comorbidities. The advent of advanced fetal imaging technologies has dramatically improved the ability to diagnose TAC in utero, allowing for early intervention, comprehensive parental counseling, and better perinatal outcomes. This discussion delves into the critical importance of prenatal diagnosis, the role of advanced imaging and genetic testing, in utero risk stratification, and the impact of early diagnosis on postnatal outcomes, while also addressing limitations in current research and future directions for improving care.

The prenatal diagnosis of TAC is critically important for several reasons. First and foremost, it enables healthcare providers to plan and coordinate care that begins at the moment of birth, which is crucial given the severity of TAC and the immediate need for medical intervention. Early and accurate prenatal diagnosis, primarily through advanced fetal echocardiography, allows for the identification of TAC’s complex anatomy, including the precise nature of the truncal valve and the origin of the coronary arteries. Such detailed anatomical information is invaluable for surgical planning and timing, ensuring that the necessary resources are available immediately after birth.

Recent advancements in fetal imaging have significantly enhanced the accuracy of diagnosing TAC in utero. Three-dimensional (3D) and four-dimensional (4D) echocardiography can be helpful in the diagnosis of TAC and its subtyping as well as in differentiation from pulmonary atresia with ventricular septal defect and the very rare proximal aorto-pulmonary window. These modalities enable the comprehensive assessment of the truncal valve, pulmonary arteries, and associated anomalies, such as interrupted aortic arch, which is present in a significant proportion of TAC cases.

Furthermore, the integration of fetal magnetic resonance imaging (MRI) into diagnostic protocols has provided an additional layer of precision. Fetal MRI, with its superior soft tissue contrast and spatial resolution, complements echocardiography by offering clearer images of extracardiac structures and the great vessels, which is particularly useful in cases where echocardiographic imaging is limited by fetal position or maternal factors. This multimodal approach to imaging is now considered the gold standard in prenatal diagnosis of complex congenital heart defects like TAC, allowing for detailed anatomical assessment and better outcome predictions.

The ability to diagnose TAC prenatally also has profound implications for delivery planning. When TAC is identified in utero, the delivery can be planned at a tertiary care center equipped with specialized neonatal and cardiac care units. This ensures that the neonate has immediate access to the necessary interventions, including prostaglandin therapy to maintain ductal patency and early surgical repair. Without prenatal diagnosis, infants with TAC may present with severe symptoms such as heart failure or cyanosis shortly after birth, requiring emergent and often suboptimal care. By contrast, a planned delivery in a controlled environment allows for the timely initiation of life-saving treatments and reduces the risk of complications associated with delayed diagnosis and intervention.

Prenatal diagnosis also plays a crucial role in parental counseling and psychological preparation. Knowing the diagnosis before birth allows parents to receive comprehensive information about the condition, the expected course of treatment, and the potential outcomes. This counseling process, ideally conducted by a multidisciplinary team that includes pediatric cardiologists, cardiac surgeons, geneticists, and neonatologists, helps parents understand the severity of the condition, the likelihood of associated genetic anomalies, and the long-term implications for their child. Early diagnosis also provides families with the opportunity to make informed decisions about the pregnancy and to prepare emotionally and logistically for the intensive care that their newborn will require.

### 4.1. Risk Stratification in Utero

In utero risk stratification is a critical aspect of managing TAC, as it provides essential information for guiding perinatal care and planning postnatal interventions. TAC is a highly heterogeneous condition, and certain subtypes and associated anatomical features are known to carry a worse prognosis. Therefore, early identification of high-risk cases allows for more tailored and intensive monitoring, both before and after birth. Previous investigations have established that moderate to severe truncal valve regurgitation or the presence of an interrupted aortic arch (IAA) are significant risk factors for mortality and postoperative complications following truncus arteriosus repair [32,33,34]. These two risk factors can already be evaluated prenatally.

One of the key aspects of in utero risk stratification is the identification of TAC subtypes. The latest classification of TAC proposed by Russell et al. based on the aortic or pulmonary dominance emphasizes the most outcome-relevant aspect of TAC [5]. Subtypes of pulmonary dominance involving complete interruption of the aortic arch (corresponds to Van Praagh A4), are associated with the highest risk. This subtype not only requires more complex surgical repair but also carries a higher risk of perioperative complications and mortality [32,33,34,35]. Older and more recent studies found that neonates with Type A4 TAC had significantly higher rates of postoperative complications, including prolonged ventilatory support, increased duration of intensive care, and higher mortality rates compared to other subtypes, even if in recent years, the postoperative outcome appears to be similar to that of children with TAC without IAA [36,37].

Another critical factor in risk stratification is the presence of truncal valve dysfunction, which significantly impacts prognosis. Prenatally, severe truncal valve regurgitation or stenosis can rarely lead to congestive heart failure already in utero with the occurrence of hydrops fetalis and, more frequently, to poor fetal growth, all of which are associated with increased perinatal mortality. The degree of truncal valve dysfunction is often a decisive factor in determining the timing of delivery and the urgency of postnatal surgical intervention and the most important factor for the outcomes of surgical repair. Occurrence and progression of truncal valve insufficiency in late second- and third-trimester pregnancy are possible. A comprehensive assessment of the truncal valve during the postnatal period is crucial in establishing a realistic prognosis for the infant’s outcome. A recent meta-analysis demonstrated a pooled overall mortality of 28.0% after TAC repair among patients with significant truncal valve insufficiency (RR of 1.70 (95% CI [1.27–2.28], *p* < 0.001) compared to neonates with competent truncal valve. Severe and moderate truncal valve insufficiency was also significantly associated with an increased risk for early mortality (RR 2.04; 95% CI [1.36–3.06], *p* < 0.001) and truncal valve reoperation (RR 3.90; 95% CI [1.40–10.90], *p* = 0.010) [38]. In their analysis, Laux et al. showed that severe and moderate regurgitation in the fetal period is also relevant postnatally, but that some newborns with a competent valve in the fetal period still develop moderate or severe insufficiency after birth before surgical repair [23]. Even if the number of fetuses with truncal valve insufficiency in the prenatal studies and this meta-analysis is too small to be able to draw significant conclusions, it seems pathophysiologically plausible that a relevant truncal valve insufficiency persists after birth and is, therefore, a risk factor for a poorer postnatal outcome.

However, interrupted aortic arch and truncal vale insufficiency occur in a relatively small subset of patients. Data from the Society of Thoracic Surgeons Congenital Heart Surgery Database (STS-CHSD) indicate that, between 2000 and 2009, a total of 572 patients underwent surgical correction of truncus arteriosus. Of these, 22 patients received concomitant truncal valve repair, 34 underwent simultaneous repair of IAA, and 5 underwent repair of both lesions. In the remaining 511 patients, 45 deaths (9%) were recorded. This suggests that early mortality remains a significant concern in pediatric patients with truncus arteriosus, even in the absence of coexisting IAA or truncal valve surgery [39].

In addition to anatomical risk factors, genetic abnormalities play a significant role in the prognosis and management of TAC. The strong association between TAC and the 22q11.2 microdeletion, which occurs in approximately 25% of cases and is linked to DiGeorge syndrome, underscores the importance of comprehensive genetic evaluation as part of prenatal care [7,40]. This genetic anomaly is associated with a spectrum of clinical features, including immune deficiency, hypocalcemia, and developmental delays, all of which can complicate the management of TAC. But even after surgical repair, the presence of a microdeletion 22q11.2 appears to be an independent risk factor for increased late mortality, usually within the first year [41].

Other risk factors for increased mortality and morbidity in newborns with TAC are a smaller right ventricle to pulmonary artery conduit size [42], but also large-sized conduits [39] as well as the need for tracheostomy [41], a birth weight below 3 kg, cardiac arrest, extracorporeal membrane oxygenation, acute kidney injury, and cardiac catheterization [43]. On the other hand, the material of right ventricular outflow tract (RVOT) conduits, commonly bioprosthetic conduits such as bovine jugular vein conduit or porcine-valved conduit, homograft, and polytetrafluoroethylene (PTFE) conduits, does not seem to influence the outcome [39,44,45]. All conduits of different materials need to be stretched and/or replaced at some point.

Anomalies of the coronary arteries are observed in up to 20% of TAC-affected neonates [31] and are sometimes regarded as a risk factor for a poorer outcome and intra- and postoperative complications [46]. The location and course of these arteries are highly variable. However, the diagnosis of anomalies of the coronary arteries in TAC is currently not possible prenatally by echocardiography, even if the outflow and in some cases the course of these arteries can be visualized in the third trimester under favorable conditions [47].

### 4.2. Integration of Genetic Counseling

Advanced genetic testing techniques, such as array comparative genomic hybridization (aCGH) and next-generation sequencing (NGS), are increasingly used to detect smaller chromosomal and genetic abnormalities that routine karyotyping might miss. These techniques are essential for identifying other chromosomal anomalies associated with TAC, such as trisomies and structural chromosomal defects, but also single gene mutations. Early identification of these genetic factors allows for more intensive prenatal monitoring, better preparation for the associated comorbidities, and the development of targeted intervention strategies.

Given the significant genetic component of TAC, integrating genetic counseling into the prenatal care of affected pregnancies is essential. Genetic counseling provides parents with critical information about the potential genetic causes of TAC, the likelihood of recurrence in future pregnancies, and the implications of associated genetic syndromes. This information is vital for helping parents make informed decisions about the pregnancy and preparing them for the possible outcomes. In cases where a genetic syndrome like DiGeorge is identified, prenatal counseling can also include discussions about the long-term care and support that the child may require.

### 4.3. Impact of Prenatal Diagnosis on Postnatal Outcomes

The outcomes of TAC have historically been poor, with high morbidity and mortality rates, particularly in the neonatal period. However, advances in surgical techniques and perioperative care over the past two decades have significantly improved survival rates. Early surgical intervention is indicated to correct the underlying cardiac defect, and studies have shown that the timing of this intervention is crucial, with earlier surgeries in the second or third week of life generally leading to better outcomes.

Prenatal diagnosis plays a critical role in improving these outcomes by allowing for meticulous planning and preparation. When TAC is diagnosed prenatally, a multidisciplinary team can coordinate care to ensure that the neonate receives the best possible start to life. This includes planning for immediate postnatal stabilization, initiation of prostaglandin therapy to maintain ductal patency, and scheduling early surgical repair, typically within the first few days of life. In cases where TAC is diagnosed prenatally, surgery is typically planned for within the first week of life to prevent complications such as heart failure, pulmonary overcirculation, and end-organ damage. The surgical approach generally involves reconstruction of the pulmonary arteries, repair or replacement of the truncal valve if necessary, and correction of associated anomalies such as interrupted aortic arch. Advances in surgical techniques, including the use of homografts and the development of more refined cardiopulmonary bypass strategies, have significantly improved the survival rates and long-term outcomes for these patients [48].

While the immediate postoperative management does not differ significantly between prenatally and postnatally diagnosed cases, early diagnosis facilitates better preparation and potentially improves short-term outcomes. However, the long-term outcomes for patients with TAC remain an area of ongoing research [41]. Although surgical techniques have improved, patients with TAC often require lifelong follow-up due to the risk of complications such as conduit stenosis, truncal valve dysfunction, arrhythmias as well as one or several reoperations or catheter interventions. Despite this, adult survivors with TAC seem to have the same quality of life as their peers in the control group [49].

Despite these advancements, several research gaps remain. Many studies on TAC are limited by small sample sizes and short follow-up periods, making it difficult to draw definitive conclusions about long-term outcomes. Additionally, the psychosocial impact of living with TAC has not been extensively studied. Future research should focus on conducting larger, multicenter studies that provide a more comprehensive understanding of the variability in outcomes associated with TAC. There is also a pressing need for long-term follow-up studies that track patients diagnosed with TAC from infancy through adulthood, examining not only their cardiac function but also their psychosocial well-being and quality of life. Such studies should investigate the long-term impact of surgical interventions, the need for reoperations, and the effects of associated genetic syndromes on overall health and development.

### 4.4. Limitations

Most of the studies in this systematic review were conducted at centers where fetuses and newborns with congenital heart defects are treated nationwide. Referral to these centers is strongly dependent on the respective organization of screening for fetal anomalies and aneuploidies. This varies between countries and has also changed considerably over the last two decades. Relevant factors for the prenatal diagnosis and outcome of fetal TAC include the extension of the basic cardiac scan from the four-chamber view alone to the additional visualization of the two outflow tracts with the great arteries both in the second trimester and now also in the first-trimester examination. Moreover, the expansion of nuchal translucency screening has facilitated the earlier diagnosis of aneuploidies and heart defects. Additionally, the introduction of cell-free DNA screening in maternal blood (NIPT) enables the detection of trisomy 21, 18, and 13 as well as microdeletion 22q11. All this, as well as the improved quality of equipment, influences the proportion of fetuses with aneuploidies and extracardiac anomalies, the timing and accuracy of diagnosis, and the proportion of terminations in the collective ultimately assigned to a center for perinatal cardiology.

One of the primary limitations in the current body of research on TAC is the challenge of conducting meta-analyses that include a sufficient number of studies with large, diverse populations. Many of the existing studies are based on small cohorts, which limits the generalizability of the findings. Additionally, the extraction of individual case data is often not feasible, which further complicates efforts to conduct comprehensive analyses. This underscores the need for larger, prospective studies that can provide more robust data on the incidence, outcomes, and management of TAC.

Another significant limitation is the reliance on routine chromosome analysis, which may overlook small chromosomal abnormalities such as microdeletions. This has likely led to an underestimation of the incidence of genetic abnormalities associated with TAC. The use of molecular cytogenetic methods, such as aCGH and NGS, is still not widespread in all centers, which limits the ability to detect and understand the full spectrum of genetic factors involved in TAC. Expanding the use of these advanced genetic techniques in prenatal diagnosis would likely provide a more complete picture of the genetic underpinnings of TAC and improve the accuracy of risk stratification.

Finally, there is a lack of long-term outcome data for individuals with TAC, particularly those diagnosed prenatally. While some studies have reported promising long-term survival rates, the data on functional outcomes, quality of life, and psychosocial impact remain sparse. Long-term studies that follow patients from prenatal diagnosis through childhood and into adulthood are urgently needed to provide a more comprehensive understanding of the outcomes associated with TAC and to guide future management strategies.

## 5. Conclusions

Truncus arteriosus communis (TAC) remains a rare and complex congenital heart defect that presents significant challenges in prenatal diagnosis, management, and postnatal outcomes. Advances in prenatal imaging, particularly fetal echocardiography and fetal MRI, have greatly improved the ability to diagnose TAC accurately in utero, enabling early intervention and comprehensive parental counseling. The integration of advanced genetic testing into prenatal care has further enhanced the ability to identify associated genetic anomalies, allowing for more precise risk stratification and better-informed decision-making.

Management strategies during pregnancy should focus on monitoring fetal growth and cardiac function, with delivery planning involving a multidisciplinary approach to ensure immediate postnatal stabilization and access to specialized cardiac care. Early surgical intervention is critical for improving outcomes, although the condition remains associated with significant risks and the potential for long-term complications. Continued research, particularly long-term follow-up studies, is essential to provide comprehensive information on the outcomes of children diagnosed with TAC and to guide future management strategies.

Overall, a coordinated, multidisciplinary approach from prenatal diagnosis through postnatal care is essential for improving the outcomes of individuals with TAC. Continued advancements in medical and surgical care, coupled with ongoing research, will be crucial in addressing the challenges associated with this complex congenital heart defect and in improving the prognosis and quality of life for affected individuals.

### What Does This Study Add to the Clinical Work

This study significantly enhances clinical practice for managing truncus arteriosus communis (TAC) by emphasizing accurate prenatal diagnosis through advanced fetal echocardiography, facilitating comprehensive parental counseling, and meticulous delivery planning in specialized centers. It provides a framework for proactive prenatal management, stressing the importance of regular monitoring to assess defect progression and associated anomalies. The study underscores the need for multidisciplinary coordination in delivery planning, ensuring immediate access to specialized neonatal and cardiothoracic surgical services.

It advocates for early surgical intervention within the first few weeks of life to prevent irreversible damage to the pulmonary vasculature, detailing procedures to repair the ventricular septal defect and separate the pulmonary arteries from the truncus arteriosus. Additionally, it outlines effective postnatal stabilization protocols, including the use of prostaglandin E1 to maintain ductal patency. These insights collectively enhance the prognosis and quality of life for individuals with TAC by promoting a comprehensive, coordinated care approach.

## Figures and Tables

**Table 1:** Quality assessment for the conduct and report of case series according to Murad et al. [8]: Guiding questions have been adjusted to research questions to assess the methodological quality of the included case series. One or two stars (*) can be awarded in the categories depending on the number of criteria that potentially need to be fulfilled.

Items	Evaluation Questions
Selection *	Do the patients represent the whole experience of the center? Is the selection method clear and does it ensure that all cases of suspected TAC are reported?
Ascertainment **	Was the presence of a suspected or confirmed TAC adequately ascertained? Was the outcome of TAC-suspected cases adequately ascertained?
Causality *	Was follow-up long enough for the evaluation of the outcome of TAC-suspected pregnancies?
Reporting *	Was the study’s approach with regard to inclusion and analysis criteria sufficiently described to allow the case series to be replicated by other researchers or for practitioners to draw relevant conclusions for their own practice?

**Table 2:** Outcome of included prenatally diagnosed local TAC cases.

	Prenatal Diagnosis	GA at Referral	Pregnancy Outcome	Postnatal Diagnosis	Surgical Outcome	Microdeletion 22q11	Associated Cardiac	Anomalies Extracardiac/Pregnancy-Related	Survival at Last Follow-Up
1	TAC type 2	22 + 2	Elective CD	TAC type 1	Primary repair	-	RAA, ASD 2, PDA, PLSVC	IUGR anal atresia with vestibular fistula	alive (23 m)
2	TAC type 1	22 + 0	Emergency CD	TAC type 1	Post-OP-NND after pulmonary artery banding	negative	Truncal valve insufficiency, PFO, myocardial hypertrophy, single coronary ostium	Polyhydramnios	NND (8 d)
3	TAC type 1	16 + 2	TOP	-	-	-	Truncal valve insufficiency and stenosis, mitral and tricuspid insufficiency,	Hypoplastic thymus	TOP
4	TAC type 1	23 + 3	TOP	-	-	negative	Hypoplastic left ventricle	missing left tibia, bilateral clubfeet, thickened left thumb, short nasal bone, prefrontal skin edema, thickened neck	TOP
5	TAC type 1	21 + 5	Elective CD	Pulmonary atresia with VSD	-	-	-	-	-
6	TAC type 1	19 + 0	SVD	TAC type 1	Primary repair	-	-	Polyhydramnios	alive (21 m)
7	TAC type 1	17 + 0	TOP	-	-	-	Hypoplastic left ventricle, mitral atresia with VSD	Cerebral ventriculomegaly, micrognathia	TOP
8	TAC type 1	24 + 6	Elective re-CD	TAC type 1	Primary repair	negative	-	-	alive (1 m)
9	TAC type 1	20 + 5	VD after IoL *	DORV	-	-	-	-	-
10	TAC type 1	20 + 3	VAVD *	TAC type 1	Primary repair	-	RAA	Penile hypospadias	alive (44 m)
11	TAC type 1	19 + 4	TOP	-	-	negative [Trisomy 13]	Overriding aorta, VSD	Alobular holoprosencephaly, microcephaly, enlarged cisterna magna, bilateral microphthalmy, proboscis, cleft lip and palate, bilateral nephromegaly, bilateral oligodactyly of the feet, bilateral polydactyly of the hands	TOP
12	TAC type 3	33 + 6	Elective CD	Pulmonary atresia with VSD (with one great right sided MAPCA arising from DAO and perfusion of the LPA via DA from the aortic arch)	-	-	RAA	-	-
13	TAC type 2	23 + 4	SVD	TAC type 2	Primary repair	negative	RAA	-	alive (33 m)
14	TAC type 2	28 + 3	VD after IoL *	TAC type 2	Primary repair	-	Peripheral pulmonary stenosis	-	alive (46 m)

* Introduction was performed with misoprostol; atrial septal defect [ASD], cesarean delivery [CD], double outlet right ventricle [DORV], induction of labor [IoL], interrupted aortic arch [IAA], neonatal death [NND], patent ductus arteriosus [PDA], persistent foramen ovale [PFO], persistent left superior vena cava [PLSVC], right aortic arch [RAA], spontaneous vaginal delivery [SVD], termination of pregnancy [TOP], vacuum-assisted vaginal delivery [VAVD], vaginal delivery [VD], ventricular septal defect [VSD].

**Table 3:** Overview of the included case series regarding methodological quality, study characteristics, and the number of included cases. Canada [CA], China [CN], France [FR], Germany [GER], intrauterine fetal death [IUFD], Italy [IT], La Réunion [RE], Netherlands [NL], prenatal diagnoses [pre], postnatal diagnoses [post], Republic of South Korea [KR], Rumania [RO], Spain [ES], termination of pregnancy [TOP], Ukraine [UK], United Kingdom [UK], United States of America [USA]. One or two stars (*) can be awarded in the categories of methodological quality depending on the number of criteria that potentially need to be fulfilled.

Study	Current Study	Abel et al. [13]	Bourdial et al. [16]	Cox et al. [17]	Duke et al. [18]	Evans et al. [19]	Galindo et al. [20]	Gómez et al. [21]	Korsuize et al. [22]	Laux et al. [23]	Lee et al. [24]	Mărgi-nean et al. [25]	Morgan et al. [26]	Sivanan-dam et al. [27]	Su et al. [28]	Swanson et al. [9]	v. Nissel-rooji et al. [29]	Volpe et al. [30]	Wolter et al. [31]
Methodological quality																			
Selection *		*	*	*	-	*	*	*	*	*	*	*	*	*	(*)	*	*	*	*
Ascertainment **		**	**	**	**	**	**	**	**	**	**	**	**	**	**	*	**	**	**
Causality *		*	*	*	*	*	*	*	*	*	*	*	*	*	(*)	-	*	*	*
Reporting *		*	(*)	*	*	(*)	*	*	*	*	*	-	*	*	-	-	*	*	*
Study characteristics																			
Country	GER	GER	RE	USA	UK	USA	ES	ES	NL	FR	KR	RO	CA	USA	CN	CA	NL	IT	GER, UA
Collection period	2019–2024	2010–2018	2002–2007	2010–2020	1990–1999	2006–2021	2005–2009	2006–2013	2005–2020	2011–2019	2003–2012	2009–2017	1990–2014	1994–2003	2017–2022	1992–2007	2002–2016	1993–2002	2008–2021
Initial diagnosis	pre	pre	pre + post	pre	pre	pre + post	pre + post	pre	pre + post	pre + post	pre	pre	pre + post	pre	pre	pre + post	pre	pre	pre
Included cases																			
Prenatal	11	34	16	23	17	32	11	10	19	66	12	17	49	11	108	43	38	23	47
Postnatal	-	-	6	-	-	7	2	-	7	16	-	-	116	-	-	93	-	-	-
Total cases	11	34	22	23	17	39	13	10	26	82	12	17	165	11	108	136	38	23	47
Diagnostic accuracy																			
TAC diagnosis	79%	88%	no data	no data	87%	33%/93% ^#^	79%	80%	68%	84%	71%	no data	6%/45% *	100%	no data	88%	82%	96%	no data
TAC subtype	86%	91%	no data	no data	no data	no data	no data	100%	92%	80%	no data	59%	no data	100%	no data	no data	no data	100%	100%
Pregnancy outcome of prenatal diagnoses																			
GA at referral ([weeks])	22 (16–33)	22 (13–28)	-	24 ± 6.3	22 (18–32)	-	24 (12–40)	20 (15–37)	-	26	24 (21–34)	24 (15–36)	22 (16–35)	27 (16–35)	23 (13–37)	-	20 (16–34)	25 (17–36)	22 (12–37)
TOP	4 (36%)	14 (41%)	14 (87.5%)	4 (17%)	4 (24%)	2 (6%)	5 (36%)	9 (75%)	2 (11%)	9 (12%)	4 (33%)	8 (47%)	11 (22%)	1 (9%)	66 (61%)	17 (40%)	18 (48%)	8 (35%)	10 (21%)
IUFD	0	1 (3%)	0	2 (11%)	0	2 (7%)	2 (22%)	0 (0)	1 (6%)	0 (0)	0	-	1 (23%)	-	9 (8%)	2 (5%)	2 (10)	2 (9%)	2 (4%)
Live births	7 (64%)	19 (56%)	2 (12.5%)	18 (74%)	13 (76%)	28 (88%)	6 (43%)	3 (25%)	10 (53%)	62 (79%)	8 (67%)	4 (24%)	37 (75%)	9 (82%)	33 (31%)	24 (56%)	18 (42%)	13 (57%)	35 (75%)

# for the study periods 2006–2009 vs. 2010–2021; * for the study periods 1990–1999 vs. 2000–2014.

**Table 4:** Cases of isolated TAC with prenatal truncal valve dysplasia and findings of postnatal echocardiographic assessment.

Study	Case No°	TAC Subtype	Prenatal Dysplasia	Postnatal Dysplasia	TV Regurgitation		TV Stenosis		Surgery	Outcome
					Prenatal	Postnatal	Prenatal	Postnatal		
Duke et al. [18]	4	I	+	+	-	+	+	+	yes	postOP-NND
	7	I	+	+	+	+	+	+	no	preOP-NND
	8	I	+	-	+	+	-	+	yes	alive
Gomez et al. [21]	3	I	+	/	+	/	-	-	/	TOP
	5	I	+	/	+	/	-	-	/	TOP
Lee et al. [24]	1	I	+	+	+	+	+	+	yes	alive
	4	I	+	+	+	+	+	+	yes	alive
	5	I	+	+	+	+	+	+	yes	alive
	8	I	+	+	+	+	+	+	yes	alive
	12	I	+	/	+	/	+	/	/	TOP
Van Nisselrooji et al. [29]	14	/	+	/	+	/	+	/	/	TOP
	16	/	+	/	+	?	-	/	/	TOP
	19	/	+	/	+	/	-	/	/	IUFD
	20	/	+	/	+	/	-	/	/	IUFD
	36	/	+	?	-	?	+	?	yes	alive
Volpe et al. [30]	6	I	+	+	+	+	+	+	no	preOP-NND
	9	I	+	+	-	-	+	+	yes	alive
	15	I	+	+	+	+	+	+	yes	postOP-NND

+ positive finding; - negative finding, s/ no information (because of intrauterine fetal demise [IUFD] or termination of pregnancy [TOP]); ? missing data.

**Table 5:** Associated anomalies and genetic analysis of patients with TAC.

Study	Current study	Abel et al.	Bourdial et al.	Cox et al.	Duke et al.	Evans et al.	Galindo et al.	Gómez et al.	Kor-suize et al.	Laux et al.	Lee et al.	Mărgi-nean et al.	Morgan et al.	Sivanan-dam et al.	Su et al.	Swanson et al.	v. Nissel-rooji et al.	Volpe et al.	Wolter et al.
**Isolated TAC**	5 (45%)	9 (26%)	-	-	6 (35%)	-	6 (46%)	4 (50%)	-	67 (82%)	9 (75%)	7 (41%)	-	-	22 (20%)	-	15 (39%)	11 (48%)	6 (13%)
**Cardiac anomalies**																			
**Truncal valve defects (%)**	2 (18%)	8 (23%)	-	?	8 (47%)	1 † (3%)	-	2 (25%)	8 (42%)	35 (61%)	8 (67%)	3 (18%)	64 (39%)	-	-	32 (29%)	9 (24%)	9 (39%)	-
**Stenosis**	1 (9%)	-	-	9 (47%)	5 (29%)	-	-	0	1 (5%)	12 (21%)	-	0	28 (17%)	-	-	13 (12%)	3 (8%)	5 (22%)	8 (17%)
**Regurgitation**	2 (18%)	-	-	9 (47%)	3 (18%)	1 † (3%)	-	2 (25%)	7 (37%)	23 (41%)	-	3 (18%)	36 (22%)	-	-	19 (17%)	6 (16%)	4 (17%)	14 (30%)
**Right aortic arch**	3 (27%)	6 (18%)	-	?	-	-	-	1 (13%)	4 (21%)	11 (13%)	2 (17%)	-	44 (27%)	-	9 (8%)	34 (30%)	8 (21%)	3 (13%)	4 (9%)
**Extracardiac anomalies**	6 (55%)	26 (76%)	-	10 (43%)	4 (24%)	-	7 (54%)	4 (50%)	-	15 (18%)	2 (17%)	5 (29%)	-	3 (38%)	86 (80%)	20 (18%)	17 (45%)	10 (43%)	17 (36%)
**Genetic testing**																			
**Tested cases**	6 (43%)	34 (100%)	-	21 (91%)	10 (59%)	35 (90%)	13 (100%)	10 (100%)	14 (54%)	78 (95%)	12 (100%)	4 (24%)	129 (78%)	-	-	68 (50%)	38 (100%)	22 (96%)	37 (78%)
Number of genetic anomalies in total (% *)	1 (17%)	13 (38%)	-	12 (57%)	-	16 (45%)	4(30%)	4 (40%)	3 (21%)	39 (50%)	2 (17%)	0	51 (40%)	-	-	-	14 (37%)	8 (36%)	15 (41%)
**Microdeletion** 22q11 (% *)	0	6 (18%)	4 (25%)	4 (19%)	3 (30%)	11 (31%)	2 (15%)	1 (10%)	1 (7%)	18 (23%)	0	0	39 (30%)	-	-	32 (47%)	7 (18%)	6 (27%)	9 (24%)
Others (% *)	1 (17%)	7 (21%)	-	8 (38%)	-	5 (14%)	2 (15%)	3 (30%)	2 (14%)	21 (27%)	2 (17%)	0	12 (9%)	-	-	-	7 (18%)	2 (9%)	6 (16%)

† at least, * proportion of number of cases who underwent genetic testing.

**Table 6:** Postnatal management and survival of prenatal and postnatal TAC cases.

Study	Current Study	Abel et al.	Bourdial et al.	Cox et al.	Duke et al.	Evans et al.	Galindo et al.	Gómez et al.	Korsuize et al.	Laux et al.	Lee et al.	Mărgi-nean et al.	Morgan et al.	Sivanan-dam et al.	Su et al.	Swanson et al.	v. Nissel-rooji et al.	Volpe et al.	Wolter et al.
Live births	7	19	8	17	13	35	8	3	16	78	8	4	165	8	16	117	18	13	35
pre-OP NND	1 (14%)	0 (0)	-	2 (11%)	5 (38%)	1 (3%)	2 (25%)	1 (33%)	1 (6%)	11 (14%)	1 (13%)	0	16 (10%)	0	-	4 (3%)	3 (17%)	3 (23%)	3 (9%)
Surgeries in total	7 (100%)	14 (74%)	-	15 (88%)	8 (62%)	34 (97%)	6 (75%)	1 (100%)	16 (100%)	67 (86%)	7 (88%)	3 (75%)	137 (83.03)	-	-	113 (96,5%)	15 (83%)	10 (77%)	32 (91%)
Corrective Surgery	6 (86%)	13 (93%)	-	15 (100%)	7 (88%)	26 (76%)	-	1 (100%)	16 (100%)	64 (96%)	7 (100%)	3 (100%)	137 (100%)	-	-	113 (100%)	15 (100%)	6 (60%)	32 (100%)
Palliative Care or Surgery	1 (14%)	0	-	1 (6%)	1 (12%)	8 (24%)	-	0	0	3 (4%)	0	1 (25%)	0	-	-	0	0	2 (20%)	0
Awaiting Surgery	0	1 (7%)	-	0	0	0	-	0	0	-	0	0	0	-	-	0	0	2 (20%)	0
Peri- and post-OP-NND	1 (14%)	5 (38%)	-	1 (7%)	3 (38%)	2 (6%)	1 (17%)	0	0	-	1 (14%)	2 (66%)	25 (18%)	1 (12%)	-	11 (10%)	1 (7%)	2 (25%)	5 (16%)
CHD	0	0	-	-	1 (8%)	1 (3%)	2 (25%)	0	0	-	0	0	12 (7%)	-	-	-	2 (11%)	-	-
Intention-to-treat-survival	6/7 (86%)	14/20 (70%)	-	14/17 (82%)	5/12 (42%)	24/26 (92%)	3/?	1/1 (100%)	16/16 (100%)	16/64 (25%)	6/8 (75%)	1/3 (33%)	39/130 (30%)	-	-	-	12/16 (75%)	7/8 (88%)	27/32 (84%)
Overall survival	6/11 (55%)	14/20 (70%)	-	14/23 (93%)	5/13 (38%)	31/35 (89%)	3/8 (38%)	1/3 (33%)	16/16 (100%)	16/78 (21%)	6/8 (75%)	1/17 (6%)	43/165 (26%)	7/8 (88%)	16/108 (15%)	-	12/18 (67%)	7/13 (54%)	27/47 (57%)
Follow-up (mean, range) [mo.]	24 (0.3–46)	42 (6–104)	-	-	41 (1–75)	- (?–67)	60 (12–144)	0.5 (0.3–0.6)	64.8 (0.1–150)	- (?–72)	12 (1–105)	4 (2–8)	103.2 (0.1–236)	- (?–1)	-	-	72 (24–120)	10	51.5

Live births [LB], preoperative neonatal deaths [pre-OP-NND], postoperative neonatal deaths [post-OP NND], childhood deaths [CHD], alive at the time of last follow-up [survivors], months [mo.]. Surgeries in total [%] = Cases undergoing surgery/LB. Primary repair [%] = Primary repair/Surgeries in total. Palliative surgeries [%] = Palliative surgeries/Surgeries in total. CHD [%] = CHD/LB. pre-OP-NND [%] = pre-OP-NND/LB. per- and post-OP-NND [%] = peri– and post-OP-NND/performed surgeries in total. survivors [%] = survivors/LB.3.11. Survival of Prenatal Diagnosed Cases.

**Table 7:** Outcome analysis of prenatal diagnosed TAC cases.

Authors	Current Study	Abel et al.	Cox et al.	Duke et al.	Gómez et al.	Korsuize et al.	Lee et al.	Mărgi-nean et al.	Sivan-danam et al.	Swanson et al.	v. Nissel-rooji et al.	Volpe et al.	Wolter et al.
Prenatal Diagnoses	11	34	23	17	10	19	12	17	11	43	38	23	47
Live births	7 (64%)	19 (56%)	17 (74%)	13 (76%)	1 (10%)	10 (53%)	8 (67%)	4 (24%)	8 (73%)	19 (44%)	18 (47%)	13 (57%)	35 (74%)
NND/CHD	1 (14%)	5 (26%)	3 (18%)	8 (62%)	0	0	2 (25%)	3 (75%)	1 (9%)	8 (42%)	6 (33%)	5 (38%)	8
pre-OP-NND	0	0	2 (11%)	5	0	0	1	0	0	2	3	3	3 (9%)
post-OP-NND	1	5	1 (7%)	3	0	0	1	2	1	4	1	2	5 (16%)
CHD	0	0	0	1	0	0	0	0	0	2	2	0	0
Surgeries total	7 (100%)	14 (74%)	15 (88%)	8 (62%)	1 (100%)	10 (100%)	7 (88%)	3 (75%)	-	17 (89%)	15 (83%)	10 (77%)	32 (91%)
Corrective Surgery	6	13	15	7	1	10	7	3 (100%)	-	17	16	6	32 (100%)
Palliative Care	1	0	0	1	0	0	0	0	-	0	0	2	0
Awaiting	0	1	0	0	0	0	0	0	-	0	0	2	0
Intention-to- treat-survival	6/7 (86%)	14/20 (70%)	14/17 (82%)	5/12 (42%)	1/1 (100%)	10/10 (100%)	6/8 (75%)	1/3 (33%)	7/8 (88%)	11/19 (58%)	12/16 (75%)	7/8 (88%)	27/32 (84%)
Overall survival	6/11 (55%)	14/34 (41%)	14/23 (93%)	5/17 (29%)	1/10 (10%)	10/19 (53%)	6/12 (50%)	1/17 (6%)	7/11 (64%)	11/43 (26%)	12/18 (67%)	8/23 (35%)	27/47 (57%)

Neonatal death [NND], childhood deaths [CHD]. Live births (%) = live births/prenatal diagnoses. NND (%) = NND/live births. Surgeries (%) = Surgeries/live births. Intention-to-treat-survival (%) = Surviving neonates/repairing surgeries. Overall survival (%) = Surviving children/prenatal diagnoses.

## Data Availability

The data that substantiate the findings of this systematic literature review are accessible within the original publications that are referenced throughout the review.
